# Cost-effective recruitment methods for a large randomised trial in people with diabetes: A Study of Cardiovascular Events iN Diabetes (ASCEND)

**DOI:** 10.1186/s13063-016-1354-9

**Published:** 2016-06-13

**Authors:** Theingi Aung, Richard Haynes, Jill Barton, Jolyon Cox, Aleksandra Murawska, Kevin Murphy, Michael Lay, Jane Armitage, Louise Bowman, ASCEND Study Collaborative Group

**Affiliations:** Nuffield Department of Population Health (NDPH), Clinical Trial Service Unit & Epidemiological Studies Unit (CTSU), Richard Doll Building, Old Road Campus, Oxford, OX3 7LF UK; Royal Berkshire NHS Foundation Trust, Reading, UK

**Keywords:** Aspirin, Omega-3 fatty acids, Diabetes, Cardiovascular disease, Randomised controlled trial, Recruitment methodology

## Abstract

**Background:**

Clinical trials require cost-effective methods for identifying, randomising, and following large numbers of people in order to generate reliable evidence. ASCEND (A Study of Cardiovascular Events iN Diabetes) is a randomised ‘2 × 2 factorial design’ study of aspirin and omega-3 fatty acid supplements for the primary prevention of cardiovascular events in people with diabetes; this study used central disease registers and a mail-based approach to identify, randomise, and follow 15,000 people. In collaboration with UK consultants and general practitioners (GPs), researchers identified potentially eligible people with diabetes from centrally held registers (e.g. for retinopathy screening) and GP-held disease registers. Permission was obtained under section 251 of the National Health Service Act 2006 (previously section 60 of the NHS act 2001) to allow invitation letters to be generated centrally in the name of the holder of the register. In addition, with the collaboration of the National Institutes for Health Research (NIHR) Diabetes and Primary Care Research Networks (DRN and PCRN), general practices sent pre-assembled invitation packs to people with a diagnosis of diabetes. Invitation packs included a cover letter, screening questionnaire (with consent form), information leaflet, and a Freepost envelope. Eligible patients entered a 2-month, pre-randomisation, run-in phase on placebo tablets and were only randomised if they completed a randomisation form and remained willing and eligible at the end of the run-in. Follow-up is ongoing, using mail-based approaches that are being supplemented by central registry data.

**Results:**

Information on approximately 600,000 people listed on 58 centrally held diabetes registers was obtained, and 300,188 potentially eligible patients were invited to join the study. In addition, 785 GP practices mailed invitations to 120,875 patients. A further 2,340 potential study participants were identified via other routes. In total, 423,403 people with diabetes were invited to take part; 26,462 entered the 2-month, pre-randomisation, run-in phase; and 15,480 were randomised.

**Conclusion:**

If sufficient numbers of potentially eligible patients can be identified centrally and the trial treatments do not require participants to attend clinics, the recruitment and follow-up of patients by mail is feasible and cost-effective. Wider use of these methods could allow more, large, randomised trials to be undertaken successfully and cost-effectively.

**Trial registration:**

Current Controlled Trials, ISRCTN60635500, registered on 14 July 2005

**Electronic supplementary material:**

The online version of this article (doi:10.1186/s13063-016-1354-9) contains supplementary material, which is available to authorized users.

## Background

Randomised controlled trials are the cornerstone for reliably evaluating the safety and efficacy of therapeutic strategies [1]. For chronic conditions, where many treatments are expected to have only moderate effects, trials need to be large in size and long in duration to achieve sufficient statistical power and ensure a robust result. The regulations surrounding clinical trials are becoming increasingly burdensome [2, 3], and as a result, the cost and complexity of a standard approach to evaluating therapies is prohibitive (typically at least £3–400 M for large clinical outcome trials), and the model is unsustainable [4]. The development of potentially effective drugs is often stopped prematurely on financial, rather than scientific grounds, and it has become more difficult to do academic trials of important scientific questions; this difficulty has resulted in the distortion of the scientific agenda.

Clinical trials are typically undertaken in a clinic-based setting either in primary or secondary care, and the recruitment of large numbers of participants may require many sites, resulting in organisational complexity and very substantial costs [4]. However, for interventions that require no ongoing physical or laboratory safety monitoring, conducting the trial by mail offers a cost-effective alternative. Several large, successful, randomised trials have been conducted using both a mailed drug supply and follow-up [5–8]. Experience from these studies shows that, with appropriate attention to the wording of the information leaflets, consent forms and questionnaires, good response rates and compliance can be achieved, and reliable information about medical events, gathered. However, previous trials had been conducted among healthcare professionals (i.e. doctors or nurses), and it was not known if such mail-based approaches to clinical trials would be feasible and acceptable in people without such a background.

ASCEND (A Study of Cardiovascular Events iN Diabetes) is a 2 × 2 factorial design randomised study to assess whether aspirin 100 mg daily versus placebo and separately, omega-3 fatty acids 1 g daily versus placebo, reduce the risk of cardiovascular events in individuals with diabetes who do not already have diagnosed occlusive arterial disease, and whether any such benefits outweigh any hazards from bleeding. To minimise costs sufficiently to allow ASCEND to be funded by non-commercial sources, the study was designed to be run mainly by mail with back-up from a 24-hour Freefone service. The rationale and design are available on the study website (http://www.ctsu.ox.ac.uk/ascend/further_pro.htm). This report describes the highly cost-effective mail-based-recruitment methods, which allowed the randomisation of 15,480 people with diabetes from around the UK into ASCEND, making it one of the largest ever trials in this patient group.

## Methods

### Trial coordination and approvals

The University of Oxford’s Clinical Trial Service Unit & Epidemiological Studies Unit (CTSU) is coordinating the study and has overall responsibility for the administration and management of the study under the guidance of a Trial Steering Committee. The University of Oxford is the regulatory sponsor of the trial. After the study had secured initial funding from the British Heart Foundation and a commitment to provide packaged aspirin and matching placebo tablets from Bayer Pharmaceuticals and omega-3 fatty acid capsules and matching placebo capsules from Solvay Pharmaceuticals (subsequently Abbott and now Mylan), Multi-centre Research Ethics Committee (MREC) approval was obtained in 2003 (North West REC, ref 03/8/087) for the study protocol and, in particular, to use centrally held diabetes registers to identify potential participants. Since local doctors were not directly involved in recruitment, the MREC approval indicated that local ethics committees need only be informed of the study, and site-specific approval was not required. Regulatory approval was obtained from the Medicines and Healthcare Products Regulatory Agency (MHRA), and permission to obtain identifiable details of people with diabetes without their explicit consent (in order to invite them to participate in the trial) was obtained from the Patient Information Advisory Group (PIAG), constituted under Section 60 of the NHS Act 2001 (subsequently the National Information Governance Board under section 251 of the National Health Service Act 2006, and more recently the Confidentiality Advisory Group). The coordinating centre ensured that the necessary Research Governance approvals were also in place for the invitations to be sent from general practices.

### Identification of participants

**Fig. 1:**
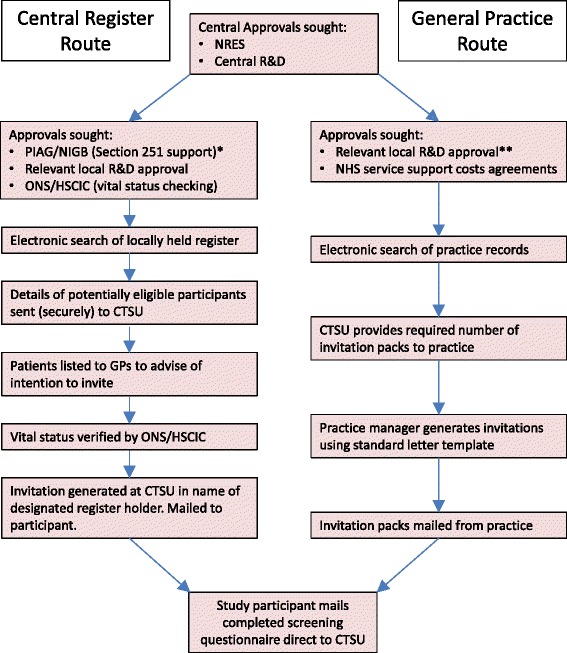
Main routes of identification and invitation of potential study participants. * At the time of recruitment for ASCEND, PIAG/NIGB approval was a separate application process. More recently it has become integrated with the central IRAS system.** ASCEND sought local R&D from every primary care trust (PCT) in England, health board in Scotland and local health board in Wales [13]. NRES: National Research Ethics Service (now part of the Health Research Authority); PIAG: Patient Information Advisory Group; NIGB: National Information Governance Board; ONS: Office for National Statistics; HSCIC: Health and Social Care Information Centre

People with diabetes were identified from two main sources: (1) centrally held diabetes registers and (2) general practice diabetes registers. Once potentially eligible individuals had responded to their invitation, subsequent processes were identical for each route of identification (Fig. 1).

#### Centrally held registers

Consultant diabetologist physicians and other relevant doctors from around the UK were invited to collaborate with the investigators in Oxford in order to allow invitation of potentially suitable individuals with diabetes from their locally held diabetes registers (such as those held for retinopathy screening). To streamline the invitation process, and in accordance with the PIAG approval, the individuals’ contact details, date of birth, and GP details were sought electronically, and lists were sent to the coordinating centre. Prior to contacting anyone, lists of potential invitees were sent to the relevant GP asking that they inform the coordinating centre if they did not wish their patients (either specific individuals or all potentially eligible patients) to be contacted about the study. No response from the GP after a reminder letter was taken as agreement to contact the patients. Immediately prior to the invitation being sent, the vital status of the person was checked with the Office for National Statistics (subsequently Health and Social Care Information Centre), to help avoid inadvertent invitation of people who had died (although delays in the availability of up-to-date information could not prevent this entirely). Large-scale, automated, mailing systems were used to generate individualised invitation letters, which were sent with the computer-generated signature of the designated holder of the diabetes register. The invitation pack included the signed cover letter, a screening questionnaire (including the consent form), the study patient information leaflet and a Freepost return envelope addressed to the coordinating centre (see Additional files 1 and 2). A 24-hour Freefone telephone service was available for trial-related enquiries from both potential participants and medical staff.

#### General practice registers

Consultants and other collaborators were also asked to identify 20–30 local GPs with computerised disease registers and to seek their agreement to mail invitations to potentially eligible individuals. In addition, the National Institutes for Health Research (NIHR) Diabetes Research Network (DRN) and the NIHR Primary Care Research Network (PCRN) identified other interested general practices and provided support for practice staff in the recruitment process. Staff in collaborating practices performed an electronic search on their practice database for potentially eligible patients. Having reviewed the list generated by this search to remove anyone considered unsuitable for the trial, they informed the coordinating centre of the number of invitation packs required, and these were sent to the practice. At the practice, an approved invitation letter was mail-merged with the patient’s name and address onto practice headed paper, and these invitation letters were added to the invitation packs provided by the coordinating centre and mailed. For practices identified via the DRN and PCRN, local network support funding was available to help with these administrative tasks.

#### Other identification routes

Other potentially eligible patients with diabetes were identified from among participants in the Medical Research Council/British Heart Foundation (MRC/BHF) Heart Protection Study (HPS) [9], and they were sent similar invitation packs, with the cover letter adapted accordingly. In addition hospital-based collaborators were sent pre-assembled invitation packs that they could hand to potentially eligible patients seen in their outpatient clinics, and randomised participants had the option of recommending a friend or relative they thought might be eligible and interested in participating in the study. With their friend’s or relative’s permission, their contact details were sent to the coordinating centre, and an invitation pack was mailed directly to the individual. The study website also facilitated the registration of potential volunteer participants. Diabetes UK, the UK patient, healthcare professional, and research charity, published a brief article about the study in their patient magazine ‘Balance’, which resulted in a number of ‘self-referrals’.

#### Method of recruitment

People were potentially eligible if aged over 40 years, had type 1 or 2 diabetes, and were not thought to have occlusive vascular disease. Preliminary eligibility was based on information provided on the completed screening questionnaire (i.e. confirmation of diabetes diagnosis, no reported history of diagnosed occlusive arterial disease, no contraindication to regular aspirin, and signed consent to participate – see Additional file 1). Completed screening questionnaires were returned (Freepost) to the coordinating centre where they were logged and then scanned using optical character recognition software to facilitate the efficient transfer of information into the study databases. Bespoke computer programs were used to validate the data, with study administrators, nurses, or clinicians performing additional checks where needed or contacting participants for clarification of responses if necessary.

#### Consent and pre-randomisation run-in

**Fig. 2:**
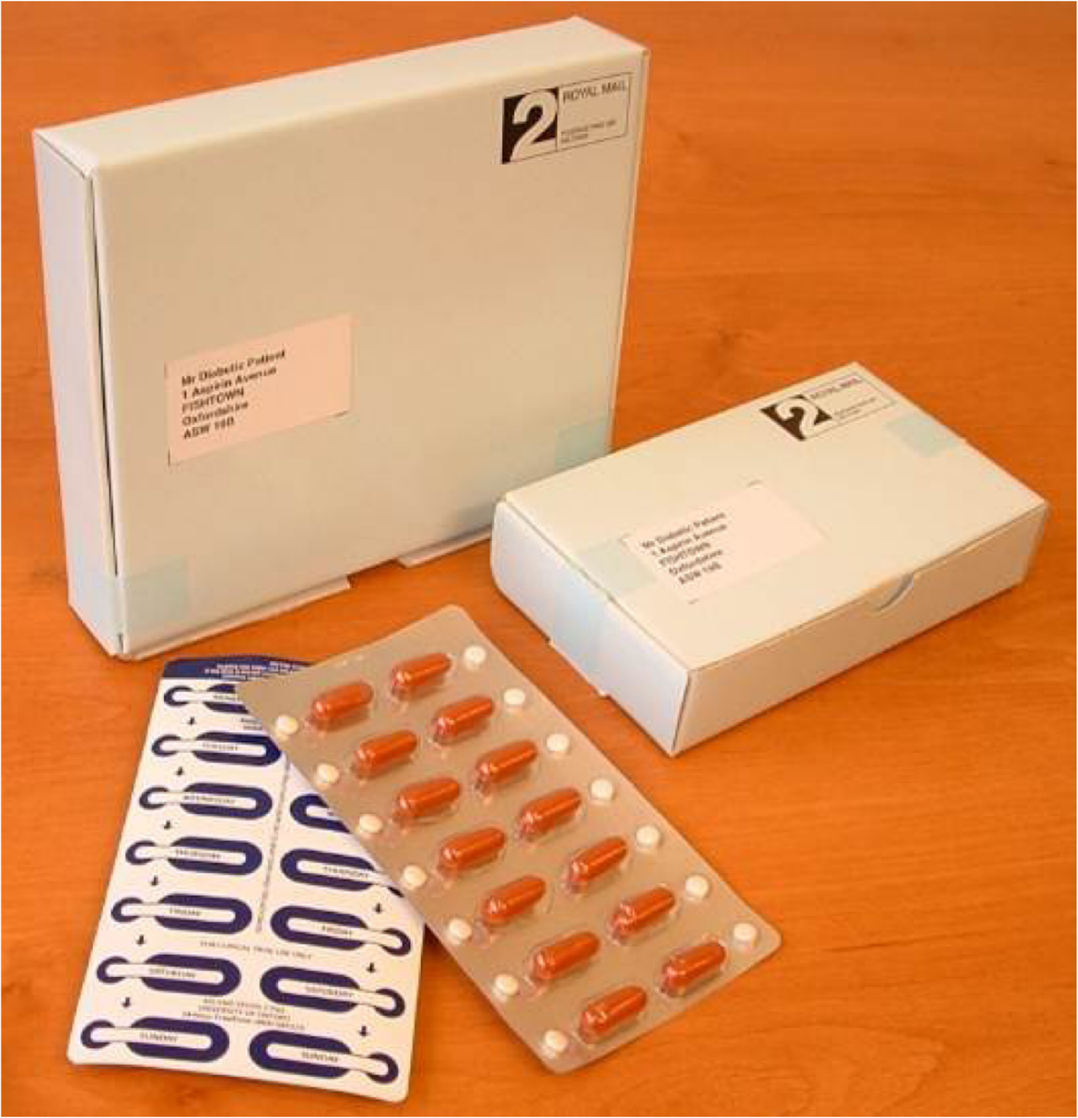
Packaged study drugs for mail-based trial

The screening questionnaire included specific questions related to consent (Additional file 1), which the participants had to sign to confirm that they had understood, and that if they had any questions, these had been addressed by study staff. The 24-hour Freefone service was available if they had any questions about the trial or wished to speak to a doctor about their involvement. During the day, this was manned by the study team, and outside working hours, a clinician was available via a radio pager. Based on the screening questionnaire responses, willing and eligible patients, all of whom had provided signed informed consent, entered a 2-month, placebo, run-in phase (single-blind) and were mailed a pre-randomisation ‘run-in’ pack of medication, which contained 8-weeks of placebo aspirin and placebo omega-3 fatty acids (Fig. 2). An information sheet about the medication was provided (including a list of contraindicated medications – see Additional file 3), along with a copy of the scanned image of their signed agreement to participate.

The purpose of the run-in was to check that patients would take the study medication and return the questionnaires regularly, thereby aiming to increase the chance that, if randomised, they would remain compliant and complete follow-up [10]. The run-in also provided the time and opportunity for the coordinating centre to inform the GP of their patient’s provisional agreement to enter the study, with an option for the GP to advise against it if they wished, and for the coordinating centre to send a blood and urine kit (see below) to the participant to obtain baseline biological samples.

**Fig. 3:**
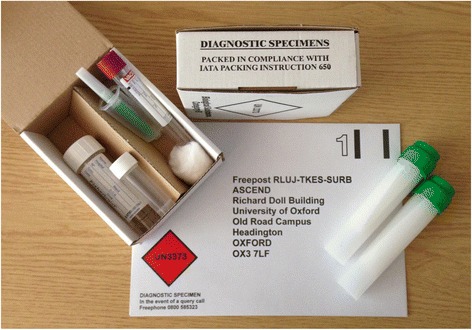
Blood and urine sampling kit for mail-based trial

During the run-in phase, a blood and urine sampling kit was sent with a supplementary information leaflet and consent form (Fig. 3 and Additional file 4). Participants were asked to take this kit to their general practice for sample collection and the samples were then mailed to the central laboratory in the containers provided. With the exception of those practices in which phlebotomy services were very limited, this approach was widely accepted, and most practices agreed to provide this service without requesting additional payment. The practice nurse was also asked to record the patient’s blood pressure and height and weight on the form provided. This allowed minimised randomisation by relevant biochemical prognostic variables (e.g. lipids, HbA1c) as well as the collection of samples for long-term storage and future analyses (including DNA).

Approximately 2 months after entering the run-in, the participants were sent a randomisation questionnaire to confirm their eligibility, collect more detail about their diabetes and current medications, and check their compliance with the study treatments during the run-in period (Additional file 5). Participants were randomised if they completed and returned a randomisation form and remained willing and eligible to participate.

## Results

A total of 423,403 potentially eligible individuals were invited via the different routes, of which, 29 % (121,254 people) returned the screening questionnaires to the coordinating centre (Table 1). Approximately one-third of those returning the questionnaire agreed to join the trial. After review of the questionnaire data, 26,462 participants (6 % of those originally invited) were willing and eligible to join ASCEND and entered the 2-month run-in period.

**Table 1:** Recruitment by route of identification

	Central registers	General practitioner (GP) registers	Others^a^	Total (% of those invited)
Invited	300,188	120,875^b^	2340	423,403
Returned valid screening form	100,563	19,478	1213	121,254 (29 %)
Entered run-in	16,091	9739	632	26,462 (6 %)
Sent randomisation form	13,481	8541	557	22,579 (5 %)
Randomised (% of those invited)	9013 (3 %)	6037 (5 %)	430 (18 %)	15,480 (4 %)

^a^MRC/BHF Heart Protection Study/self-referral/Friends & Family referral/consultant clinic invitations

^b^Based on number of screening forms sent by coordinating centre to GP practices to be mailed to participants

Randomisation questionnaires were sent to 22,579 patients. Of these, 15,480 people returned a completed questionnaire, remained willing and eligible to participate, and were randomised into ASCEND using a computer-based minimisation algorithm. Approximately 40 % of all patients who entered the run-in dropped out before randomisation, and half of these (approximately 5500 participants) had no clinical reason to stop the trial but simply declined to continue. Overall, 4 % of those invited were randomised: 3 % from centrally held registers (9013 patients) and 5 % from GP registers (6037 patients).

**Fig. 4:**
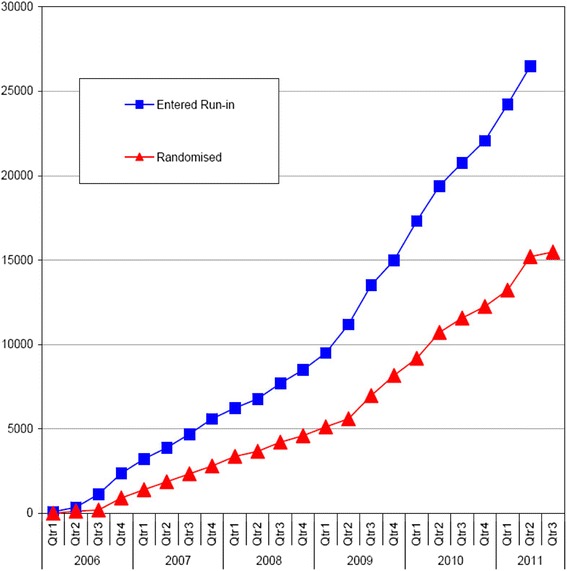
Cumulative recruitment of study participants by year

The recruitment process took longer than expected (Fig. 4) but accelerated after mid-2009 due to both the increased availability of the large central registry data (regional retinopathy registers) and to the support from the DRN and PCRN. More than 700 general practices helped with recruitment for the study, from which approximately 6000 of the randomised patients were identified. The majority of practices were identified with the help of the networks, whose support of ASCEND resulted in more than 5000 participants being recruited into the study (Table 2).

**Table 2:** Number of GP practices identified and participant recruitment via the Primary Care Research Network (PCRN), Diabetes Research Network (DRN) and other routes

	Number of practices recruited	Number of patients invited	Number of patients entered run-in	Number of patients randomised (% of those invited)
PCRN	512	79,471	6733	4207 (5 %)
DRN	156	24,420	1772	1065 (4 %)
Other	117	16,984	1234	765 (5 %)
Total	785	120,875	9739	6037 (5 %)

If a completed screening questionnaire was not received within approximately 2 weeks of the initial invitation from a centrally held register, a reminder questionnaire was sent. Approximately one-fifth (38,785 of 203,083) of those who received a reminder returned either the original or the reminder screening questionnaire. Similarly, a reminder was sent if a randomisation questionnaire was not returned within 2 weeks. Approximately two-thirds (3110 of 5101) of those to whom randomisation questionnaire reminders were sent, replied, and this led to 2183 patients being randomised. Overall, nearly half (4111 of 9013) of all randomised patients recruited via the centrally held register route were sent a reminder for at least one of the questionnaires.

The availability of information (e.g. sex, date of birth, and post code) from the centrally held registers allows the response rate to the invitation to be compared among the different types of people (Table 3). Younger invitees were more likely to express an interest in participating in the study, even if they were not eligible based on their returned screening form (14 % of those < 50 years old vs 7 % of those ≥ 70 years old, trend *p* value < 0.0001). However, amongst people who were eligible for the trial and entered the run-in phase, the percent randomised did not vary substantially according to age. There was also a slightly better initial response from men than women (11 % vs 8 %, chi-square *p* < 0.0001), but when those who were ineligible at screening are taken into account, the proportion randomised of those entering run-in was similar by sex (56.2 % men vs 55.5 % women, chi-square *p* = 0.4) (Table 3).

**Table 3:** Response to invitation; entering pre-randomisation run-in phase; and randomised by age, sex, and Townsend Index (central register route only)

		No. invited	Responded and willing to participate^a^ (% of invited)	Entered run-in (% of invited)	Randomised (% of invited)	Percent of those entering run-in who are subsequently randomised
Age (years)^b^						
	<50	12,753	1,729 (14 %)	1,262 (10 %)	694 (5 %)	55 %
	≥50, < 60	59,635	7,580 (13 %)	4,914 (8 %)	2,801 (5 %)	57 %
	≥60, < 70	93,526	11,040 (12 %)	6,103 (7 %)	3,543 (4 %)	58 %
	≥70	134,274	9,508 (7 %)	3,812 (3 %)	1,975 (1 %)	52 %
Sex						
	F	130,889	10,642 (8 %)	5,931 (5 %)	3,297 (3 %)	56 %
	M	169,299	19,215 (11 %)	10,160 (6 %)	5,716 (3 %)	56 %
Townsend Index^c^						
	< -3	64,054	7,635 (12 %)	4,649 (7 %)	2,781 (4 %)	60 %
	≥ -3 < 0	100,057	10,544 (11 %)	6,022 (6 %)	3,467 (3 %)	58 %
	≥0 < 2	47,597	4,207 (9 %)	2,179 (5 %)	1,201 (3 %)	55 %
	≥2 < 4	41,932	3,576 (9 %)	1,697 (4 %)	838 (2 %)	49 %
	≥4 < 6	30,637	2,466 (8 %)	1,009 (3 %)	496 (2 %)	49 %
	≥6	15,354	1,287 (8 %)	458 (3 %)	195 (1 %)	43 %
Urban/rural location^d^						
	Urban	244,718	23,729 (10 %)	12,590 (5 %)	6,960 (3 %)	55 %
	Rural	54,116	5,923 (11 %)	3,397 (6 %)	2,004 (4 %)	59 %
	Unknown	1,354	205 (15 %)	104 (8 %)	49 (4 %)	47 %
Total						
		300,188	29,857(10 %)	16,091 (5 %)	9,013 (3 %)	56 %

^a^Includes willing but ineligible responses. Eligibility likely to vary in subgroups due to differing incidence of prior vascular disease

^b^Based on age on the date screening invitation generated

^c^Based on postcode at screening (lower values indicate least deprived). Score unknown for 557 of those invited

^d^Based on postcode at screening (using ONS 2011 Rural-Urban Classification for Small Area Geographies)

**Fig. 5:**
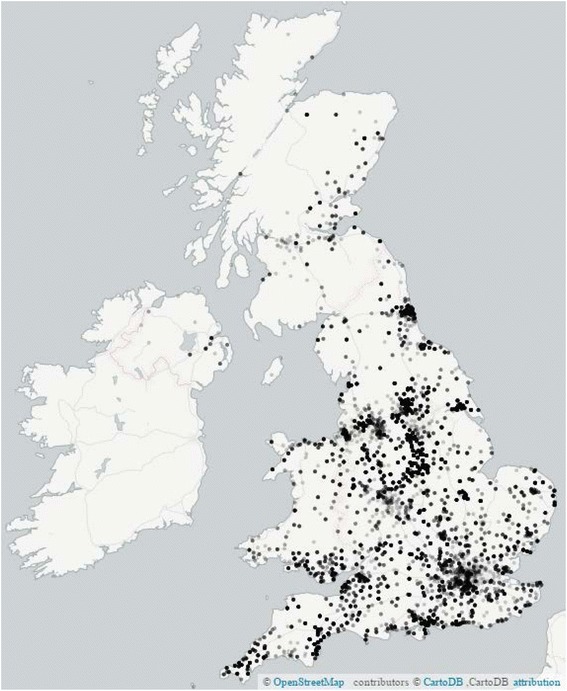
Location of randomised participants in the UK (postcode of home address)

A particular advantage of the mail-based trial methodology used in ASCEND is that with no requirement to attend study clinics, participation is not limited by geographical proximity to a study centre. Figure 5 shows the location of the home addresses of the randomised participants in ASCEND, with recruitment covering both rural and urban areas across the UK. The response to invitation was slightly greater from those living in rural areas compared with those in cities (10.9 % vs 9.7 %, *p* < 0.0001, Table 3). This is likely to be in part due to differences in the Townsend index (a measure of material deprivation based on the subject’s home post code), in which a substantial variation is observed from 12 % among those in the least deprived areas to 8 % from the most deprived areas (trend *p* value < 0.0001, Table 3). However, a highly significant rural vs urban effect still persists after allowing for Townsend index (*p* < 0.0001).

The number of days between mailing a study invitation and receipt of the response could be recorded for screening questionnaires sent by the coordinating centre and for all randomisation questionnaires. The mean (SD) time from original invitation to response was 24 (27) days and 14 (15) days for the screening and randomisation questionnaires, respectively. To keep study costs down, second class postage was used for all routine mailings, so the minimum achievable response time was, therefore, 4 days. Ninety-five percent of responses to the screening and randomisation questionnaires were received within approximately 2 months and 1 month, respectively.

A 24-hour Freefone telephone service for queries from participants or their doctors was available to support the recruitment process. Over the 6-year recruitment phase, 8800 telephone calls were logged to this service: 3500 were incoming calls with enquiries from participants or their carers/relatives or doctors, and 5300 were outgoing calls made by study staff, typically to clarify information that had been written on the screening and randomisation questionnaires. Approximately half of all telephone calls (both outgoing and incoming) related to participants who were not subsequently randomised.

Blood and urine kits were sent to 22,858 patients who entered the pre-randomisation phase and who had not informed the coordinating centre that they wished to withdraw before the kits were due to be sent. Samples (either blood or urine or both) were received by the laboratory from 13,270 individuals, among whom 11,685 were subsequently randomised. The average delay between sample collection and receipt at the coordinating centre laboratory (sent by first class post to limit sample delays) was 2 days.

## Discussion

ASCEND is one of the largest ever randomised trials among people with diabetes. It achieved its recruitment target of 15,000 participants by means of central and local diabetes registers to identify patients who might be suitable and by using highly cost-effective mail-based systems to send screening and randomisation questionnaires, provide study drugs, and collect biological samples. The trial is funded by a grant to the University of Oxford from the British Heart Foundation (£3.7 million), which covers the costs of running the study over a 15-year period (to include planning, recruitment, follow-up, and study close-out and reporting activities). Within this budget, the costs of printing and postage for the mail-based recruitment process were less than £0.5 million, which is substantially less than the clinic staff costs that would be required for a standard clinic-based approach at this large scale. Study drug and additional funding for drug packaging (£3.6 million) was provided by Bayer and Solvay Pharmaceuticals (subsequently Abbott, and now Mylan). In addition, the 668 practices identified via the DRN and PCRN were eligible for local network support funding (typically around £500 per practice) to help with recruitment activities. Overall, the total costs of this major trial are therefore an order of magnitude lower than those of a typical commercial clinic-based study (generally at least £3–400 M for large clinical outcome trials [4]).

Detailed baseline characterisation of the randomised participants has been possible from the information collected on the mailed screening and randomisation questionnaires and from mailed blood and urine samples. ASCEND illustrates that if large enough numbers of potentially eligible patients can be identified, and automated methods can be adopted, it is possible to recruit a large study population successfully by mail.

Access to centrally held registers of potentially eligible patients was crucial to the success of recruitment into ASCEND. Although this required the transfer of patient-identifiable information from the register holder to the coordinating centre without the patient’s prior consent, existing legislation allows this to be done within a strict legal framework. Without access to these registries, more than 2000 GP practices would need to have been identified and, even if this had been possible, such an approach would have increased the costs and time to recruit significantly. Previous patient volunteer focus group work conducted by CTSU for other studies has confirmed that most patients find this approach acceptable, as long as robust information governance standards are adhered to.

In ASCEND, a small number of complaints were received about the transfer of data without consent. In the majority of cases, the complainant was unaware of the relevant legislation, and a simple verbal explanation of the process was sufficient to clarify any concerns, with many such individuals subsequently agreeing to join the trial. The complainant remained dissatisfied in only 28 cases (0.01 % of 300,188 people invited from centrally held registers) and requested removal of personal data from the study database. The coordinating centre had a standard operating procedure for such requests, which were acted on promptly. Whereas previous mail-based trials had successfully recruited from populations of healthcare professionals [5–8], ASCEND has demonstrated the acceptability of this approach in a general diabetic population.

Overall, less than one-third of those invited replied to the invitation to take part, and the majority of those who did reply declined to join the study. Other groups have reviewed possible methods to improve the response to postal and electronic questionnaires in order to identify effective strategies to improve recruitment to trials and epidemiological studies. A Cochrane review reported substantial heterogeneity among trials, evaluating more than 100 different approaches to increasing the response to postal questionnaires (typically for epidemiological studies) [11]. Strategies involving monetary incentives and the use of recorded delivery of the questionnaire appeared to be successful, approximately doubling the response rate to postal questionnaires. However, such approaches would add substantially to the cost of a large trial such as ASCEND. Furthermore, those recruited by means of financial incentives might not remain adequately compliant with follow-up and study treatment in longer-term studies. Sending reminders in ASCEND was an effective strategy, which substantially improved the response rate, without which, an additional 140,000 potentially eligible people would have needed to be invited.

Follow-up in ASCEND is ongoing, using mail-based approaches supplemented by central registry data. Study participants receive requests every 6 months for follow-up information. This can be provided either by means of a paper questionnaire, by telephone to the coordinating centre, or using a web-based interface via an internet browser, according to the individual’s preference. For participants who are no longer able or willing to complete questionnaires, follow-up information is obtained from their GP. Overall, good rates of follow-up are being achieved using these cost-effective methods. At present, approximately 95 % of all live study participants have follow-up information available from within the last 12 months, and efforts are ongoing to contact those participants for whom follow-up is due.

The currently observed compliance (blinded) with the aspirin/placebo study treatment at the end of the first year post-randomisation is approximately 85 %, with a further 5–7 % decline in compliance annually thereafter. Despite participants having no routine direct contact with the study team, this is comparable to clinic-based trials in similar populations [12]. However, notably, the compliance with study treatments is somewhat lower among those who were sent a reminder randomisation form compared with those who replied to the initial mailing. For example, at the study mid-point (45 months after randomisation), 61 % of those sent a randomisation reminder were compliant with their aspirin/placebo study tablets versus 68 % of those where no reminder was sent (*p* < 0.0001). This reduction in compliance became apparent within the first 6 months after randomisation and, although reminders were essential for the completion of recruitment, the implications for reduced compliance in those who do not readily respond to questionnaire mailings is an important consideration.

On the other hand, the use of a pre-randomisation run-in is a valuable methodological tool to enhance compliance, especially in the early phase of a long-term study [10]. Of those who entered the single-blind placebo run-in period in ASCEND, approximately 40 % dropped out of the study before randomisation. Had there been no run-in, these withdrawals would probably have occurred after randomisation (most likely in the first 6–12 months), thereby substantially reducing the statistical power of the study.

Recruitment into ASCEND took longer than initially hoped as a result of a variety of factors, including research governance delays [13], the time taken to obtain the electronic records from the diabetes registers, establishing robust IT systems to monitor the study, and an increase in the original recruitment target. However the involvement of the former local NIHR Diabetes and Primary Care Research Networks across England and Scotland provided a valuable extra resource, which boosted recruitment and, had they been established sooner, might have shortened the recruitment period. A substantial infrastructure for patient recruitment to research studies continues to be available through the NIHR Clinical Research Network. The response to invitation was higher among those identified from general practice compared with those in central registries (5 % vs 3 % of those invited were eventually randomised). This may have been partly due to the ability to pre-screen potential participants to exclude those with established vascular disease but also because participants were more likely to respond positively to a GP whom they knew.

The design of ASCEND included an optional baseline blood and urine sample collection during the pre-randomisation run-in phase. This exercise was funded by a separate project grant from the British Heart Foundation (£140 k). Previous transport studies have demonstrated that a wide range of analytes (including HbA1c lipids and cystatin C as a measure of renal function) and genetic polymorphisms can be reliably measured in whole blood samples with delayed separation [14, 15]. During the past few years, extensive experience has been gained with obtaining cardiovascular risk factor measurements from mailed blood samples [16]. In ASCEND, this approach has allowed measured baseline risk factor information to be obtained from 75 % of those randomised at very low cost, which will allow the effects of the study treatments to be assessed within subgroups defined by biological measures.

## Conclusions

ASCEND is designed to be streamlined and highly cost-effective. When completed, the trial will have cost < £10 million overall. This includes both the NHS service support costs provided to general practices and the substantial costs of drug packaging and distribution (which are typically covered by the pharmaceutical industry and not usually included in the budget quoted for many large-scale outcome studies). Using the methods described, ASCEND has randomised nearly 15,500 people with diabetes, making it one of the largest ever randomised trials in this patient group. The questions it aims to address are clinically relevant for the hundreds of millions of people worldwide with diabetes and, with good follow-up and compliance, will add valuable information on the balance of benefits and risks of these treatments, including important data on the use of aspirin for cancer prevention. The strategies which helped make recruitment successful include (1) simple inclusion and exclusion criteria, (2) the central coordination of recruitment, (3) the ability to identify a large pool of potentially eligible people, and (4) the involvement of local research networks. The success of these methods in ASCEND show that, with good planning, mail-based methodology is cost-effective and could be more widely adopted for the assessment of interventions that require little monitoring.

## Additional files

Additional file 1: Example of screening questionnaire, including consent form. (PDF 167 kb) Additional file 2: Patient information leaflet. (PDF 367 kb) Additional file 3: Study treatment information leaflet. (PDF 113 kb) Additional file 4: Blood and urine sampling information leaflet. (PDF 521 kb) Additional file 5: Randomization questionnaire. (PDF 198 kb) Additional file 6: ASCEND Study Collaborative Group. (PDF 19 kb)

## Abbreviations

ASCEND: A Study of Cardiovascular Events iN Diabetes
BHF: British Heart Foundation
CTSU: Clinical Trial Service Unit
DRN: Diabetes Research Network
GP: general practitioner
MRC: Medical Research Council
PCRN: Primary Care Research Network

## References

[1] Collins R, MacMahon S (2001). Reliable assessment of the effects of treatment on mortality and major morbidity, I: clinical trials. Lancet.

[2] Califf RM (2006). Clinical trials bureaucracy: unintended consequences of well-intentioned policy. Clin Trials.

[3] Reith C, Landray M, Devereaux PJ, Bosch J, Granger CB, Baigent C (2013). Randomized clinical trials--removing unnecessary obstacles. N Engl J Med.

[4] Eisenstein EL, Collins R, Cracknell BS, Podesta O, Reid ED, Sandercock P (2008). Sensible approaches for reducing clinical trial costs. Clin Trials.

[5] Peto R, Gray R, Collins R, Wheatley K, Hennekens C, Jamrozik K, et al. Randomised trial of prophylactic daily aspirin in British male doctors. BMJ (Clin Res Ed). 1988;296:313–6. 10.1136/bmj.296.6618.313 PMC2544821 3125882

[6] Steering Committee of the Physicians' Health Study Research Group (1989). Final report on the aspirin component of the ongoing Physicians' Health Study. N Engl J Med.

[7] Rexrode KM, Lee IM, Cook NR, Hennekens CH, Buring JE (2000). Baseline characteristics of participants in the Women's Health Study. J Womens Health Gend Based Med.

[8] Christen WG, Gaziano JM, Hennekens CH (2000). Design of Physicians' Health Study II—a randomized trial of beta-carotene, vitamins E and C, and multivitamins, in prevention of cancer, cardiovascular disease, and eye disease, and review of results of completed trials. Ann Epidemiol.

[9] Heart Protection Study Collaborative Group (2003). MRC/BHF Heart Protection Study of cholesterol-lowering with simvastatin in 5963 people with diabetes: a randomised placebo-controlled trial. Lancet.

[10] Lang JM, Buring JE, Rosner B, Cook N, Hennekens CH (1991). Estimating the effect of the run-in on the power of the Physicians' Health Study. Stat Med.

[11] Edwards PJ, Roberts I, Clarke MJ, Diguiseppi C, Wentz R, Kwan I (2009). Methods to increase response to postal and electronic questionnaires. Cochrane Database Syst Rev.

[12] Belch J, MacCuish A, Campbell I, Cobbe S, Taylor R, Prescott R (2008). The prevention of progression of arterial disease and diabetes (POPADAD) trial: factorial randomised placebo controlled trial of aspirin and antioxidants in patients with diabetes and asymptomatic peripheral arterial disease. BMJ.

[13] Haynes R, Bowman L, Rahimi K, Armitage J (2010). How the NHS research governance procedures could be modified to greatly strengthen clinical research. Clin Med.

[14] Clark S, Youngman LD, Palmer A, Parish S, Peto R, Collins R (2003). Stability of plasma analytes after delayed separation of whole blood: implications for epidemiological studies. Int J Epidemiol.

[15] Peakman TC, Elliott P (2008). The UK Biobank sample handling and storage validation studies. Int J Epidemiol.

[16] Clarke R, Breeze E, Youngman L, Sherliker P, Bell P, Shah S (2000). Re-survey of the Whitehall study of London civil servants: changes in risk factors for cardiovascular disease during 29 years of follow-up. J Cardiovasc Risk.

