# Supplementary material for: Cost-effective recruitment methods for a large randomised trial in people with diabetes: A Study of Cardiovascular Events iN Diabetes (ASCEND)
Source: Trials. 2016 Jun 13;17:286. doi: 10.1186/s13063-016-1354-9 (PMC4907276; doi:10.1186/s13063-016-1354-9)
Supplement: Additional file 1: — Example of screening questionnaire, including consent form. (PDF 167 kb) [file 13063_2016_1354_MOESM1_ESM.pdf]

Gardentown **NHS**
NHS Trust

ASCEND
Clinical Trial Service Unit (CTSU)
Richard Doll Building
University of Oxford
Old Road Campus
Headington, Oxford
OX3 7LF

Office Tel: 01865 743888
Office Fax: 01865 743981
Freefone: 0800 585323
E-mail: ascend@ctsu.ox.ac.uk
Website: www.ctsu.ox.ac.uk/ascend

Ref. A797-9797

Mr Thomas White
24 Raspberry Road
Gardentown
Gardenshire
GA3 5TR

Dear Mr White,

4 July 2011

ASCEND: A Study of Cardiovascular Events iN Diabetes

I am writing to invite you to participate in the ASCEND research study of the prevention of heart attacks and strokes in people with diabetes. At this hospital we are working with Oxford University's Clinical Trial Service Unit to help identify suitable people for this nationwide study. So, we are writing (having first informed your GP, Dr Rose Gardener) to all those people on our local diabetes register who are aged over 40 and may be suitable, in order to find out whether they might be interested in taking part. The purpose of the study is to assess whether aspirin and/or naturally-occurring oils are useful for preventing heart attacks and strokes in people with diabetes who have not had circulatory problems.

Please read the enclosed Information Leaflet entitled "ASCEND: Invitation to join a large medical research project". It is then up to you whether or not you would like to take part. If you would like to, then please complete the attached questionnaire. Based on your answers, the study coordinators will write and tell you whether or not you would be suitable.

If you have any questions regarding the study you may telephone the study co-ordinators (Professor Jane Armitage or Dr Louise Bowman) on Freefone 0800 585323. Alternatively, you may wish to discuss matters with your GP or diabetes nurse before deciding whether to join. If you do not want to take part this will have no effect on your usual medical care. If you want to join the study then please complete the questionnaire and sign the Agreement to Participate. We hope you will decide to take part in ASCEND. If you do not want to participate then please indicate this on the questionnaire on the back of this letter so that you do not get approached again. In either case, please return the questionnaire in the Freepost envelope provided.

Thank you for your help.

Yours sincerely,

Dr Jack Black
Consultant Physician
Gardentown Hospital

Enc: Information Leaflet
Freepost envelope

DR [V1.13_061109] Pt ID: A123-4567

07090709

British Heart Foundation

ASCEND: Screening Questionnaire

INSTRUCTIONS FOR COMPLETION:

Please complete the questionnaire in BLOCK CAPITALS using blue or black ink.

Please place a cross in the appropriate box, e.g. Yes ☒ No ☐

(If you make a mistake, fill the entire box and mark the correct box, e.g. Yes ■ No ☒)

OR write clearly in the appropriate boxes, e.g. 2 6 / 0 1 / 2 0 0 7 (Day / Month / Year)

1. Contact Details

Please write your name and contact details clearly in the boxes provided.

Title: Mr ☐ Mrs ☐ Ms ☐ Miss ☐ Other

First name(s):

Surname:

Address:

Postcode:

Home telephone number (inc. code):

Daytime telephone number (inc. code):

2. Personal Details

Date of birth: __ / __ / 1 9 __ (Day / Month / Year) Sex: Male ☐ Female ☐

3. Joining ASCEND

Please read the enclosed leaflet **(ASCEND: Invitation to join a large medical research project)**, and indicate whether you are interested in taking part in ASCEND: Yes ☐ No ☐

If you answered **YES**, then please complete **ALL** the remaining sections of this questionnaire, sign and date the form, and return it in the FREEPOST envelope provided.

If you answered **NO**, then return the questionnaire in the FREEPOST envelope provided (but do not complete the remaining sections).

4. GP Details

Please give your GP's surname and initials, as well as the address of the GP practice.

GP surname: GP initials:

Address:

Postcode:

Need help completing this form? Please call Freefone 0800 585323

5. Medical History

5.1 Has a doctor ever told you that you had any of the following?

*Please cross **ONE** box only for each question*

a)	Diabetes, Type 1 or Type 2 (i.e. "sugar" diabetes)	Yes ☐	No ☐
b)	Heart attack	Yes ☐	No ☐
c)	Angina (chest pain from the heart)	Yes ☐	No ☐
d)	Stroke or ministroke (sometimes called TIA)	Yes ☐	No ☐
e)	Coronary artery bypass operation (CABG or "cabbage")	Yes ☐	No ☐
f)	Coronary angioplasty ("balloon", "stent" insertion or PTCA)	Yes ☐	No ☐
g)	Other arterial surgery or angioplasty (e.g. leg bypass) (Do not include angiogram) If **Yes**, please specify:	Yes ☐	No ☐
h)	Liver disease (active or chronic, or cirrhosis) If **Yes**, please specify:	Yes ☐	No ☐
i)	Cancer **within the last 5 years** (e.g. skin, breast, lung, bowel etc) If **Yes**, please give the type of cancer:	Yes ☐	No ☐
j)	Other serious illness If **Yes**, please specify:	Yes ☐	No ☐

S-240407

5.2 In the last 6 months have you been in hospital with, or has a doctor said you have:

a)	Active peptic (stomach or duodenal) ulcer?	Yes ☐	No ☐
b)	Bleeding from the stomach or bowel?	Yes ☐	No ☐

6. Current Medication

As a participant in ASCEND, you would be asked not to use NON-STUDY aspirin, medications containing aspirin or blood thinning drugs on a regular basis (i.e. more than one day per week) unless this becomes necessary.

6.1 Do you currently take any of the following regularly?

*Please cross **ONE** box only for each question*

a)	Aspirin (e.g. Anadin, Caprin, Disprin, Imazin, PostMI)	Yes ☐	No ☐
b)	Warfarin (Marevan), Acenocoumarol (Nicoumalone, Sinthrome) or Phenindione	Yes ☐	No ☐

6.2 Are you known to be allergic to aspirin or omega-3 fatty acid (fish oil) supplements? Yes ☐ No ☐

6.3 Are you willing to avoid medications containing aspirin (apart from ASCEND study treatment) during the course of the study? Yes ☐ No ☐
(N.B. you could use paracetamol instead for pain relief)

0 7 0 9 0 7 0 9

Need help completing this form? Please call Freefone 0800 585323

Please read this **Agreement to Participate**, and if you are willing then please CROSS the boxes, SIGN and DATE the form using blue or black ink, and return it in the FREEPOST envelope provided.

7. Agreement to Participate

Please cross (☒) **EVERY box** to confirm that you have read and understood the following:

☐	I have read and understood the leaflet **"ASCEND: Invitation to join a large medical research project"** ASCEND: Patient Information Leaflet [V8.4_121010]
☐	I have had an opportunity to telephone the Freefone number 0800 585323 and ask any relevant questions. All my questions have been answered to my satisfaction **OR** I decided that I did not need to ask any questions
☐	I understand that my participation in the ASCEND study is voluntary and that I am free to withdraw from the study at any time without my medical care or rights being affected
☐	I understand that information about my progress in the ASCEND study will be recorded on a computer database, and that these data will be stored securely and confidentially on a computer at Oxford University
☐	I agree that information about any serious illnesses (such as heart attacks, strokes or cancers) may be supplied in confidence to the study coordinators by my own doctors and by NHS and other central registries for use in the ASCEND study
☐	I agree that my hospital and other health records may be looked at in confidence by authorised individuals from the ASCEND study and by regulatory authorities to check the study is being carried out correctly
☐	I understand that my GP will be informed about this provisional agreement to participate in the ASCEND study, and that in about 2 months time I will have another opportunity to decide whether or not I want to join the long-term part of the study

I am happy to take part in ASCEND:

ASCEND Screening Questionnaire [V3.4_240407]

Signature: ____________ (Please use blue or black ink)

& **PRINTED** name: ____________ Today's date: __ __ / __ __ / 2 0 __ __
Day Month Year

Please check that you have answered every question, and signed and dated the form. Return the completed form in the Freepost envelope provided (no stamps needed) to:-

Freepost RLUJ-TKES-SURB, ASCEND, Richard Doll Building, University of Oxford, Old Road Campus, Headington, Oxford, OX3 7LF

If you have any questions about the study, please contact the coordinating centre in Oxford on FREEFONE: 0800 585323 (preferably during office hours 9 am - 5 pm, Monday to Friday)

If this questionnaire indicates that you are suitable to enter the preliminary part of ASCEND, a box containing ASCEND tablets (aspirin or placebo) and capsules (one or other natural oil) will be mailed to you. A copy of this Agreement to Participate, for you to keep, will also be mailed.

If the questionnaire suggests that the study medications may not be suitable for you, then we shall write and tell you.

Thank you very much
