# Supplementary material for: Cost-effective recruitment methods for a large randomised trial in people with diabetes: A Study of Cardiovascular Events iN Diabetes (ASCEND)
Source: Trials. 2016 Jun 13;17:286. doi: 10.1186/s13063-016-1354-9 (PMC4907276; doi:10.1186/s13063-016-1354-9)
Supplement: Additional file 2: — Patient information leaflet. (PDF 367 kb) [file 13063_2016_1354_MOESM2_ESM.pdf]

ASCEND

A Study of Cardiovascular Events iN Diabetes

INVITATION TO JOIN A LARGE MEDICAL RESEARCH PROJECT

A randomised study of aspirin and of natural oils for the primary prevention of cardiovascular events in diabetes

You are being invited to take part in a research study. Before you decide whether to participate, it is important for you to understand why the research is being done and what is involved. Please take time to read the following information carefully and discuss it with friends or relatives if you wish. You are entirely free to decide whether or not to take part in this trial. If you choose not to take part, the standard of care you are given by your own doctors will not be affected.

If there is anything that is not clear, or if you would like more information, please telephone the ASCEND Freefone number (0800 585323) and speak to a study nurse or doctor. The study is to be conducted mainly by mail, so no extra clinic visits will be required.

Please see reverse for a summary of the main information contained in this booklet.

ASCEND: Patient Information Leaflet [V8.4_121010]

Co-ordinated by:

Clinical Trial Service Unit, University of Oxford
E-mail: ascend@ctsu.ox.ac.uk
Website: www.ctsu.ox.ac.uk/ascend

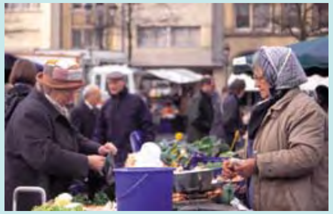
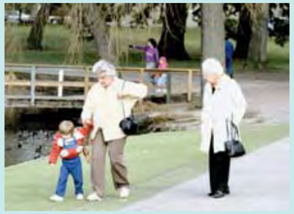
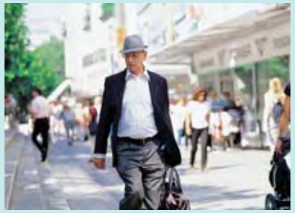

Aspirin, heart disease and strokes

Patients with diabetes may be at increased risk of developing heart disease or suffering a stroke. Aspirin prevents heart attacks and strokes in people who have existing problems with their heart or blood circulation. But it is not known whether aspirin would be helpful in people with diabetes who have not yet been diagnosed with heart or circulatory problems.

Serious (but uncommon) complications from the regular use of aspirin are bleeding in the stomach or intestinal tract. Typically this might happen in only about 1 per 1000 people taking aspirin regularly each year. Extremely rarely, aspirin may cause bleeding in the brain (about 1-2 per 10,000 people taking aspirin each year). Previous studies in people with known circulatory problems have shown that about 10 times as many people given aspirin have avoided a heart attack or stroke as have experienced a serious complication. However, in people with diabetes and no circulatory problems, it is not known whether the benefits of aspirin will outweigh the possible risks.

Omega-3 fatty acids and diabetes

Naturally occurring oils that are rich in omega-3 fatty acids (such as fish oils) may reduce the chances of a recurrent heart attack among people who have survived at least one heart attack. These oils have not been widely tested in people with diabetes, but there are reasons to hope that they may be helpful (although this is unproven). Taking regular supplements of such oils may have little or no beneficial effect among people living in a country (such as Britain) where most people eat a balanced diet. It is also possible that the long term use of these oils could, on balance, be slightly harmful – but this too is unknown.

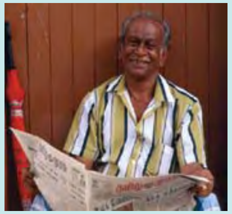
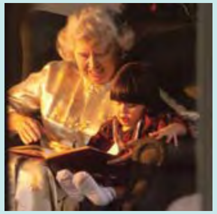
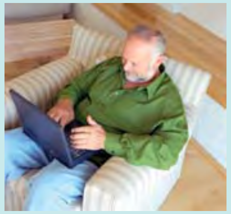
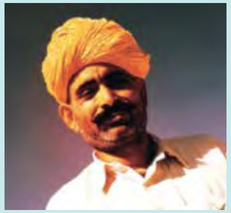

What the study hopes to answer

The main purpose of the ASCEND study is to find out whether long-term treatment with aspirin in people with diabetes, who are not already known to have heart or circulatory problems, produces benefits by preventing heart attacks and strokes which outweigh the possible risks of bleeding. ASCEND will also help establish whether oils, rich in omega-3 fatty acids, are of any importance in reducing the chances of having a heart attack in people with diabetes who have not yet got circulatory problems.

Why have I been chosen?

ASCEND will involve at least ten thousand men and women from around Britain who, like you, are being invited to take part because they have diabetes. This invitation has come from either your own GP or a local Specialist because they think you might be suitable for the study. Alternatively you may have been recommended by a friend or relative who is already taking part in the study or volunteered yourself having read about the study. It is up to you to decide whether or not to take part in this study. If you do decide to take part, you would, of course, be free to withdraw from the study treatment at any time without necessarily giving any reason (and without adversely affecting the medical care you can expect from your own doctors). In particular, at the end of the first 2 months, when you finish your first box of treatment, you will have the chance to withdraw if you have any second thoughts or problems with study treatment.

What taking part in ASCEND involves

Everyone taking part will have agreed to do so voluntarily, knowing that it may involve them in taking study treatment for at least 5 years. The daily study treatments (which would be sent to you by mail) will be a single white tablet and a single brown capsule taken from a blister pack. The white tablets will contain either active aspirin (100mg) or a similar looking inactive substance called a “placebo”. Whether or not a participant receives active or placebo tablets will be determined randomly (like tossing a coin). Each participant will have a 50% chance of receiving active aspirin and a 50% chance of receiving placebo (“dummy”) tablets. The brown capsules will each contain 1 gram of naturally occurring oil, either mainly omega-3 fatty acids or mainly olive oil.

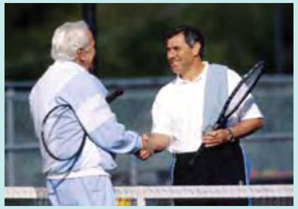

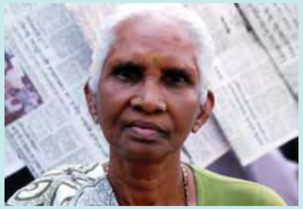

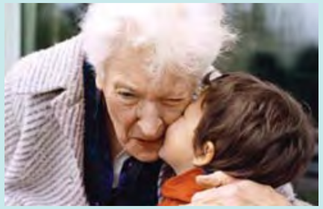

Each participant will have a 50% chance of receiving the omega-3 containing capsules and a 50% chance of receiving olive oil capsules. The type of study treatment being taken will not generally be known by you or your doctor. This information will be known only by certain staff at the coordinating centre in Oxford, but it would be made available to your doctor if this were ever medically necessary. This design helps ensure that reliable information will be obtained about the effects of these potentially important treatments.

What you have to do to join the study

If you might like to participate in this study you should complete the brief Screening Questionnaire on the inside of the letter, sign the Agreement to Participate and return them both in the enclosed Freepost envelope. We will use your answers on the questionnaire to check that you are suitable for the study. If you are suitable, then we will send a box of conveniently packaged study treatments, and ask you to start taking one tablet and one capsule each day by mouth for the next 2 months. We shall also inform your general practitioner of your involvement in the study and check that they are happy for you to continue in the study.

Within a few weeks of receiving this first pack of study treatment, you will also be sent an **optional** blood and urine sampling kit. If it is convenient for you to do so, you would be asked to attend your local surgery to have a small blood sample taken (about 2 teaspoons full) and to provide a urine specimen. Measurements of your height, weight and blood pressure would also be recorded at the surgery and this information, along with the sample, would then be mailed to the ASCEND coordinating centre.

Long-term commitment to the study

Towards the end of the 2 months you will be sent a second study questionnaire. This will allow you to indicate whether or not you would be willing to continue taking the study treatments long-term. Participation in the study does require a commitment to take the study treatments regularly for at

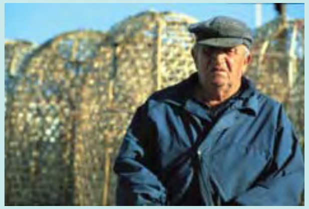

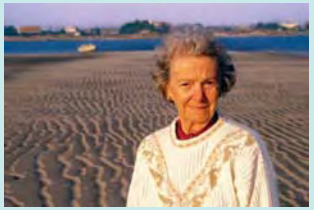

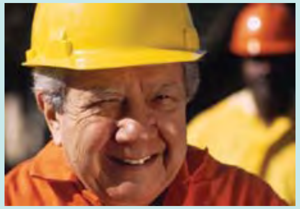

least 5 years and to complete questionnaires regularly. **If you do not think that you would be willing or able to do this then it would be better not to join in the first place**. If you decide to continue you would then be sent further supplies of the study treatments and asked to take one tablet (which would be active or dummy aspirin) and one capsule (containing one or other naturally-occurring oil) every day for the next 5 years. Further questionnaires would be sent out at 6-monthly intervals. We would ask you to tell us about your current medication and any changes to your health since your last questionnaire. Additional supplies of study treatment would be sent to you 6-monthly if you were willing to continue taking it. If you do stop during the first 2 months then no further enquiries will be made of you. But, if you decide to continue, we would like to remain in contact with you for the next several years – even if you stop taking the study treatment during this period. **Throughout the study, your own doctors would remain fully responsible for all your other medical care as usual**. However, if you develop any unexpected symptoms which you believe may be due to study treatment you should contact a study doctor on the 24-hour Freefone service: **0800 585323**.

What are the side-effects and risks of taking part?

A low dose of aspirin is being used in this study in order to minimise any stomach upset or other gastrointestinal problems. Some minor bleeding (e.g. after having blood taken) and bruising may be experienced by some people, but serious bleeding is likely to be rare. We shall monitor whether aspirin causes an unacceptable level of bleeding during the study. Bleeding risks with aspirin may be somewhat greater among those who are taking warfarin (Marevan) or other blood thinning drugs (e.g. Acenocoumarol (Nicoumalone, Sinthrome) or Phenindione). So, if you are taking any of these blood thinning drugs you would not be suitable to join the study, and if you are prescribed them later we recommend stopping the study aspirin/placebo tablets. People who join the study would be asked to avoid taking aspirin-containing painkillers, and to take an alternative (such as paracetamol) whenever pain relief is necessary. All other prescribed treatments can be taken as usual. There are no other lifestyle or dietary restrictions required.

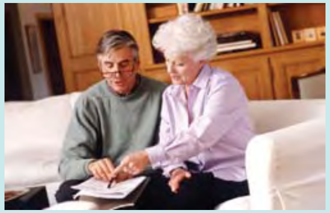
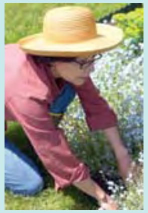
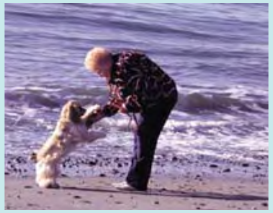
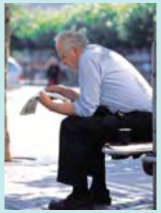

The doses of the naturally occurring oils being tested in ASCEND are not known to cause any particular problems, although some people may experience gastro-intestinal ("tummy") disturbances. If you did experience any symptoms that you thought were related to either of the study treatments, medical advice is available at all times through the 24-hour Freefone service: **0800 585323**.

What are the possible benefits of taking part?

We hope that both the study treatments may help you. However, this cannot be guaranteed. The information we get from this study may help us to treat future patients with diabetes better and may help to prevent many thousands of heart attacks and strokes.

What if new information becomes available?

Sometimes during the course of a research project, relevant new information becomes available about the treatment that is being studied. If this happens we will tell you and your general practitioner about it and you can discuss whether you want to continue in the study. A study doctor is available through the 24-hour Freefone service if either you or your GP need to discuss any new information.

What happens at the end of the study?

When the research study finishes, we will inform you and your GP of the study results. You will then be able to decide whether or not you should take aspirin and/or omega-3 fatty acids. After the study finishes we will no longer continue to provide study medication for you. But, if the study results suggest possible benefit, you could discuss with your GP whether you should take either of these treatments routinely. We will also publish the study results in a professional medical journal as soon as possible after the study finishes. You would not be identified individually in any published report.

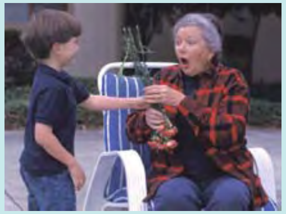
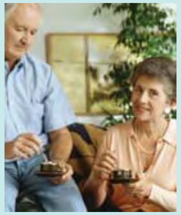
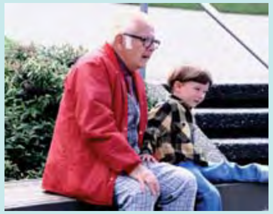
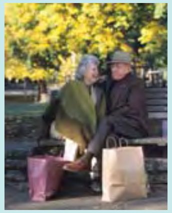

Images posed by models for illustrative purposes (© 2004 JupiterImages)

What if something goes wrong?

In the unlikely event of you being harmed as a result of taking part in the ASCEND study, the University of Oxford provides insurance cover and you would retain the same rights of care as any other patient treated in the National Health Service.

Will my taking part in this study be kept confidential?

The coordinating centre would seek information from participants' own doctors and from NHS and other central registries about any serious illnesses (such as heart attacks, strokes, cancers etc) that occur. All such information would be used, in confidence, only for medical research purposes and for routine regulatory and audit purposes.

Study organisation

The ASCEND study has been designed, and is coordinated, by Oxford University's Clinical Trial Service Unit. It involves the collaboration of many doctors and nurses around the country. The study design has been reviewed and agreed by independent Research Ethics Committees, which include people from outside the medical profession. The British Heart Foundation has provided a grant to conduct this research study, and packaged study treatment has been provided free by Bayer (makers of the aspirin/placebo) and Abbott (who are providing the natural oils). An independent Data Monitoring Committee will review various outcomes among participants during the study, and will inform the organisers if any important new information has emerged that needs to be provided to participants and their doctors.

Any questions about the study should be directed to the coordinating centre in Oxford either by telephone (24-hour Freefone service: 0800 585323) or by mail to: ASCEND Study, CTSU, Richard Doll Building, Old Road Campus, Oxford, OX3 7LF. Alternatively you can e-mail us on ascend@ctsu.ox.ac.uk

ASCEND

Summary of invitation to join a large medical research project

- Having diabetes may increase the risk of heart attacks and strokes
- Aspirin and omega-3 fatty acids benefit people who have survived a heart attack
- It is not clear whether people with diabetes who have not shown signs of circulatory problems should take aspirin or omega-3 supplements regularly
- Most people with diabetes and no circulatory problems do not take aspirin or omega-3 supplements regularly
- Low-dose aspirin is generally very safe, but does increase the risk of bleeding
- Omega-3 fatty acids at the doses being taken in ASCEND are also considered safe
- The purpose of ASCEND is to find out whether aspirin and/or omega-3 fatty acid supplementation prevents heart attacks and strokes in people with diabetes who have not shown signs of circulatory problems
- If these treatments are shown to be safe and effective for people with diabetes, then their widespread use could lead to the prevention of many thousands of heart attacks and strokes and the saving of many lives
- With your help we can answer these questions reliably with the ASCEND study

If you have any questions about the study then please feel free to contact the coordinating centre on Freefone: 0800 585323

If you think you might be interested in joining this research study please complete and return the attached questionnaire and agreement to participate. A copy of your signed agreement to participate will be returned to you when your first pack of study treatment is sent out.

Please keep this information sheet for your own records.

THANK YOU FOR YOUR HELP

Website: www.ctsu.ox.ac.uk/ascend
