# Supplementary material for: Cost-effective recruitment methods for a large randomised trial in people with diabetes: A Study of Cardiovascular Events iN Diabetes (ASCEND)
Source: Trials. 2016 Jun 13;17:286. doi: 10.1186/s13063-016-1354-9 (PMC4907276; doi:10.1186/s13063-016-1354-9)
Supplement: Additional file 3: — Study treatment information leaflet. (PDF 113 kb) [file 13063_2016_1354_MOESM3_ESM.pdf]

Below is a list of aspirin-containing medications that we request participants in ASCEND wherever possible **NOT** to take. If you require pain relief, paracetamol is a good and safe alternative which can be taken in combination with the study treatment if necessary.

**PREPARATIONS CONTAINING ASPIRIN (to be avoided if possible)**	
Alka-Seltzer	Cardio EC
Alka-Seltzer XS	Co-codaprin
Alka XS Go	Codis 500
Anacin	Disprin
Anadin	Disprin Direct
Anadin Extra	Disprin Extra
Anadin Extra Soluble	Doan's backache pills
Anadin Maximum Strength	Dristan tablets
Angettes 75	Equagesic
Apo-ASA	Entrophen
Asaphen	Extra Power
Asasantin Retard	Fynnon
Askit	Gencardia
Aspav	Imazin XL
Aspro Clear	Maximum Strength Aspro Clear
Beechams Lemon Tablets	Micropirin
Beechams Powders	Mrs Cullen's
Benoral	Novasen
Benorilate	Nurse Sykes Powders
Boots Back Pain Relief	Nu-Seals Aspirin
Boots Seltzer	Phensic
Caprin	Post MI 75 EC

IF YOU HAVE ANY QUESTIONS OR CONCERNS RELATING TO YOUR ASCEND STUDY TREATMENT PLEASE CALL FREEFONE 0800 585323

British Heart Foundation

ASCEND

A Study of **C**ardiovascular **E**vents i**N D**iabetes

UNIVERSITY OF OXFORD

A randomised study of aspirin and of natural oils for the primary prevention of cardiovascular events in diabetes

STUDY TREATMENT INFORMATION

This leaflet contains important information relating to your ASCEND study treatment. Please read all the information contained in this leaflet very carefully. If you have any questions about your study treatment, please feel free to call an ASCEND study nurse or doctor on the Freefone number 0800 585323.

Please keep this information leaflet in a safe place for future reference.

Co-ordinated by:
Clinical Trial Service Unit, University of Oxford
E-mail: ascend@ctsu.ox.ac.uk
Website: www.ctsu.ox.ac.uk/ascend

MRC Medical Research Council

1. Please check that each pack of study treatment you receive has arrived undamaged (the seals along the edge of the box should still be intact). If a treatment pack arrives damaged, or appears to have been tampered with, please contact the ASCEND office on **Freefone 0800 585323**.
2. Take one tablet and one capsule from the pack each day. It may help you remember if you take the treatment at the same time each day. Possible side effects may be reduced if you take the tablets with food.
3. If you forget to take a tablet or capsule at your usual time, you may still take it later the same day. However, if you miss a whole day or more, do not make up the missed tablets. Leave them in the calendar blister card and continue with the treatment for the next appropriate day.
4. If you reach the end of the last calendar blister card before receiving your next supply, or if you lose the study treatment, please contact the ASCEND office on **Freefone 0800 585323**.
5. Keep the medication out of the reach and sight of children, and store it in a dry place away from excessive heat, cold or moisture.
6. If you are anticipating surgery, you should stop the white study tablets (aspirin or placebo) for 10 days prior to surgery and resume upon recovery, or as soon as your doctor approves.
7. We ask participants **not** to use non-study aspirin or aspirin-containing medications wherever possible (see summary over page for a list of these). For simple pain relief paracetamol is a good alternative and can be safely taken in combination with the study medication.
8. If you experience minor symptoms that you believe may be related to the study tablets or capsules, you could try discontinuing them for a few days and then try taking them again. If you experience any major symptoms or feel you must stop the tablets or capsules altogether, then please telephone the coordinating centre on **Freefone 0800 585323**.
9. **If you are prescribed any of the following drugs by your own doctors please stop your white study tablets and telephone the coordinating centre on Freefone 0800 585323 as soon as possible to inform us:**

**Drug Names**	***Brand Names***
aspirin	See list over the page
warfarin	*Marevan*
acenocoumarol (nicoumalone)	*Sinthrome*
phenindione	*Dindevan*
clopidogrel	*Plavix*
dipyridamole	*Persantin, Persantin Retard* or *Asasantin Retard*

Removing your tablets and capsules:

1

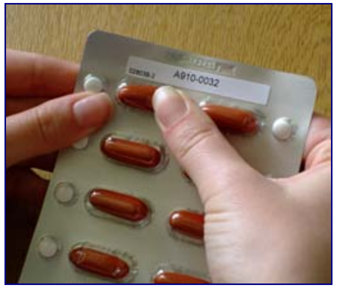

First remove the brown capsule. Apply moderate pressure towards the end of the capsule nearest the spine of the blister card.

2

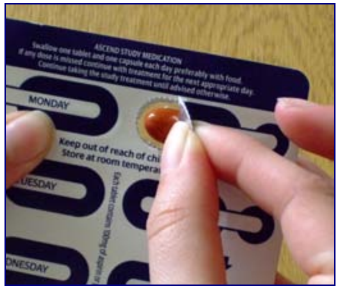

Then peel back the foil and remove the brown capsule.

3

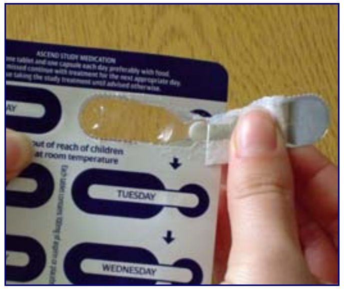

Continue to peel away the foil towards the edge of the blister card until the white tablet is partially revealed.

4

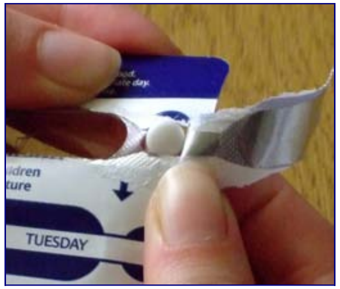

Finally, it should now be possible to push out the white tablet.
