# Supplementary material for: Cost-effective recruitment methods for a large randomised trial in people with diabetes: A Study of Cardiovascular Events iN Diabetes (ASCEND)
Source: Trials. 2016 Jun 13;17:286. doi: 10.1186/s13063-016-1354-9 (PMC4907276; doi:10.1186/s13063-016-1354-9)
Supplement: Additional file 4: — Blood and urine sampling information leaflet. (PDF 521 kb) [file 13063_2016_1354_MOESM4_ESM.pdf]

ASCEND

A Study of **C**ardiovascular **E**vents i**N** **D**iabetes

A randomised study of aspirin and of natural oils for the primary prevention of cardiovascular events in diabetes

INFORMATION ABOUT OPTIONAL BLOOD & URINE SAMPLING

This leaflet contains important information about providing an optional blood and/or urine sample for the ASCEND study. Please read all the information contained in this leaflet carefully. If you have any further questions about providing a blood and/or urine sample, please feel free to call an ASCEND study nurse or doctor on the Freefone number 0800 585323.

Please keep this information sheet for your own records.

THANK YOU FOR YOUR HELP

Co-ordinated by:
Clinical Trial Service Unit, University of Oxford
E-mail: ascend@ctsu.ox.ac.uk
Website: www.ctsu.ox.ac.uk/ascend

Blood and urine sample for immediate and future medical research

Thank you for agreeing to enter ASCEND (A Study of Cardiovascular Events iN Diabetes). With your help and that of thousands of other people, we hope to be able to answer the important questions about whether aspirin and/or omega-3 fatty acids prevent heart disease and strokes in people with diabetes who have not had any circulatory problems.

We would now like you to consider whether you would be happy to provide an **optional** blood sample and/or urine specimen, both for immediate measurements and for long-term storage. The enclosed sampling kit is to be used to collect the blood sample (about two teaspoons of blood) and urine specimen. This will then be sent to the coordinating centre laboratory, where the measurements would be made. However, you will still be eligible to continue in ASCEND even if you would prefer not to give permission for these measurements or the long-term storage of your samples.

Please take time to read the following information carefully and discuss it with your GP, friends or relatives if you wish. If there is anything that is not clear, or if you would like more information, please do not hesitate to telephone the ASCEND Freefone number (0800 585323) and speak to a study nurse or doctor.

Immediate blood and urine measurements

At the beginning of ASCEND we would like to measure certain things in the blood and urine (such as glucose control, cholesterol levels and kidney function) which we know affect the risk of heart problems and diabetes complications. Your GP would be informed of these results, so that any relevant action could be taken. These measurements allow us to see whether the effects of the treatments being used in the study vary between different types of people taking part.

What future research studies might be able to answer

Although ASCEND is aiming to discover whether aspirin and/or omega-3 fatty acids protect against heart disease and strokes in people with diabetes, there are likely to be many other causes of these conditions. Some causes, such as high blood pressure, are already known and doctors are able to treat this problem with drugs. We suspect, however, that some other factors play a role in diabetes complications, but there is limited understanding of these processes. If we were able to preserve your blood and urine samples long-term (e.g. for 20-30 years) in a very cold freezer, then future advances in medical knowledge might eventually enable us to answer some of these questions by defrosting and analysing such samples. In particular, we might analyse your blood samples in the future to look at your genes in order to study the influence of certain genes on the development of complications in diabetes. This may help us to develop new treatments for future generations of patients with diabetes.

What are the risks of allowing my samples to be stored long-term?

There are no risks at all to you. The study coordinators in Oxford University will be the only people who are able to link test results with individual participants, and the data will be held confidentially within a secure computer system. The information from any future analyses of your samples, including details of your genes, will not be provided to you, your doctors, or anybody else. In particular, having these samples stored and subsequently tested would not affect your ability to obtain insurance.

What if I don’t wish to allow my blood or urine samples to be stored long-term?

If you are happy to provide a blood and/or urine sample for immediate measurements but do not wish the sample/s to be stored for future analyses then please indicate this on the enclosed consent form by crossing “Yes” or “No” as appropriate against the different sections.

If, after reading this leaflet, you feel that you would be willing to provide a blood and/or urine sample then please complete and sign the enclosed consent form (remembering to cross (**X**) a "Yes" or "No" box for each statement).

You will then need to take the sampling kit, consent form and Freepost envelope to where you usually have blood taken, where the samples will be collected and your height, weight, blood pressure and pulse recorded. The sample(s) and completed consent form can then be sent to the ASCEND coordinating centre in the Freepost envelope provided.

If you or the nurse/phlebotomist have any further questions about this, please contact the ASCEND coordinating centre on the Freefone number 0800 585323

THANK YOU FOR YOUR HELP
