# Supplementary material for: Cost-effective recruitment methods for a large randomised trial in people with diabetes: A Study of Cardiovascular Events iN Diabetes (ASCEND)
Source: Trials. 2016 Jun 13;17:286. doi: 10.1186/s13063-016-1354-9 (PMC4907276; doi:10.1186/s13063-016-1354-9)
Supplement: Additional file 5: — Randomization questionnaire. (PDF 198 kb) [file 13063_2016_1354_MOESM5_ESM.pdf]

ASCEND
A Study of Cardiovascular Events iN Diabetes

ASCEND Document Ref. A765-4321

ASCEND
Clinical Trial Service Unit (CTSU)
Richard Doll Building
University of Oxford
Old Road Campus
Headington
Oxford
OX3 7LF

Office telephone: 01865 743888
Office fax: 01865 743981
Freefone: 0800 585323
E-mail: ascend@ctsu.ox.ac.uk
Website: www.ctsu.ox.ac.uk/ascend

Mr Thomas White
24 Raspberry Road
Gardentown
Gardenshire
GA3 5TR

12 August 2011

Dear Mr White,

ASCEND: A Study of Cardiovascular Events iN Diabetes

It is now about 2 months since you provisionally agreed to take part in ASCEND and received a box of study treatment. We would now like to know whether you are happy to continue into the long-term part of the ASCEND study. You may remember that the purpose of the study is to assess whether aspirin and/or naturally-occurring oils are useful for preventing heart attacks and strokes in people with diabetes who do not already have diagnosed circulatory problems.

On the back of this letter is a brief questionnaire which we would like you to complete and sign. Then please return it in the enclosed Freepost envelope. If you have any questions regarding the study you may telephone us or one of the other ASCEND staff on Freefone 0800 585323. Alternatively, you may wish to discuss matters with your GP or diabetes nurse before deciding whether to continue.

We hope you will decide to continue in ASCEND. If you do, then we shall send a new pack of study treatment as soon as we have received this completed form back from you. Subsequently, you will be sent a brief questionnaire to complete every six months and new supplies of study treatment. Alternatively, if you do not want to participate then please indicate this on the form and return it in the Freepost envelope provided.

Yours sincerely

Professor Jane Armitage   Dr Louise Bowman
Study Coordinators

On behalf of Dr Jack Black, Gardentown NHS Trust

Enc: FREEPOST envelope

Pt ID: A123-4567   B22-1

0 7 0 9 0 7 0 9

Medical Research Council

ASCEND: Randomisation Questionnaire

INSTRUCTIONS FOR COMPLETION:

Please complete the questionnaire in BLOCK CAPITALS using blue or black ink.

Please place a cross in the appropriate box, e.g. Yes [X] No [ ]

(If you make a mistake, fill the entire box and mark the correct box, e.g. Yes [■] No [X])

OR write clearly in the appropriate boxes, e.g. 2 6 / 0 1 / 2 0 1 0 (Day / Month / Year)

1. Contact and GP Details

Please check that the contact details for you and your GP are correct. If not, then please call Freefone 0800 585323 and provide the correct information. Please quote the reference number from the covering letter on the front of this questionnaire.

Your details:

Mr Thomas White
24 Raspberry Road
Gardentown
Gardenshire
GA3 5TR

GP details:

Dr AB Brown
The Medical Centre
Strawberry Field
Gardentown
Gardenshire
GA3 9ZZ
01234 543210

2. Continuing in ASCEND

Are you willing to continue taking study tablets and capsules in ASCEND for the next 5 years? Yes [ ] No [ ]

If you answered **YES**, then please complete **ALL** the remaining sections of this questionnaire, sign and date the form, and return it in the FREEPOST envelope provided.

If you answered **NO**, then return the questionnaire in the FREEPOST envelope provided (but do not complete the remaining sections).

3. About Your ASCEND Medication

Please indicate how regularly you have taken your ASCEND medication since you received it:

**White Tablets** (aspirin/placebo)		**Brown Capsules** (one or other natural oil)	
Every day	[ ]	Every day	[ ]
Most days	[ ]	Most days	[ ]
Only occasionally	[ ]	Only occasionally	[ ]
Never	[ ]	Never	[ ]

*Please cross **ONE** box only in **EACH** column*

4. About Your Diabetes

4.1 What **year** was your diabetes diagnosed? [ ][ ][ ][ ]

4.2 Do you use INSULIN (injections or via pump) for your diabetes? Yes [ ] No [ ]

4.3 If **Yes**, did you start insulin within one year of having diabetes? Yes [ ] No [ ]

4.4 Are you known to have diabetes changes at the back of the eye? Yes [ ] No [ ]

4.5 If **Yes**, have you ever had laser treatment to the eye for this? Yes [ ] No [ ]

4.6 Do you take treatment for high blood pressure or hypertension? Yes [ ] No [ ]

Need help completing this form? Please call Freefone 0800 585323

5. Confirming Your Medical History

5.1 Has a doctor ever told you that you had any of the following?

*Please cross **ONE** box only for each question*

a) Heart attack Yes [ ] No [ ]

b) Angina (chest pain from the heart) Yes [ ] No [ ]

c) Stroke or ministroke (sometimes called TIA) Yes [ ] No [ ]

d) Coronary artery bypass operation (CABG or "cabbage") Yes [ ] No [ ]

e) Coronary angioplasty ("balloon", "stent" insertion or PTCA) Yes [ ] No [ ]

f) Other arterial surgery or angioplasty (e.g. leg bypass) (Do not include angiogram) Yes [ ] No [ ]

If **Yes**, please specify: ____

g) Liver disease (active or chronic, or cirrhosis) Yes [ ] No [ ]

If **Yes**, please specify: ____

h) Cancer **within the last 5 years** (e.g. skin, breast, lung, bowel etc) Yes [ ] No [ ]

If **Yes**, please give the type of cancer: ____

i) Other serious illness Yes [ ] No [ ]

If **Yes**, please specify: ____

R-190510

5.2 In the last 6 months have you been in hospital with, or has a doctor said you have:

a) Active peptic (stomach or duodenal) ulcer? Yes [ ] No [ ]

b) Bleeding from the stomach or bowel? Yes [ ] No [ ]

6. About You

6.1 Please give your date of birth: [ ][ ] / [ ][ ] / 1 9 [ ][ ] (Day / Month / Year)

0 7 0 9 0 7 0 9

6.2 Which best describes your ethnic origin?

White [ ] Black: African/Caribbean [ ]

Indian, Pakistani or Bangladeshi [ ] Other [ ]

*Please cross **ONE** box only*

6.3 Please provide your **weight** in light indoor clothes without shoes (round to nearest whole number): [ ][ ][ ] kgs **OR** [ ][ ] stones & [ ][ ] lbs

6.4 Please provide your standing **height** without shoes (round to nearest whole number): [ ][ ][ ] cms **OR** [ ] feet & [ ][ ] inches

6.5 Do you smoke cigarettes regularly (i.e. on most days)? Yes [ ] No [ ] If **Yes**, give approximate number smoked per day: [ ][ ]

6.6 If **No**, have you **ever** smoked regularly? Yes [ ] No [ ] If **Yes**, give the age you stopped: [ ][ ] years

7. Current Medication

7.1 Please list your current medication as **prescribed by your doctor** (names only, doses not required). Please **do not** list any medication (e.g. aspirin) you have stopped to enter ASCEND.

....

....

....

7.2 Please list any other treatments you take regularly (i.e. more than twice a week), for example, pain killers, vitamins, supplements, over-the-counter tablets or capsules.

....

....

8. Alternative Contact

It would be very helpful for us if you could provide the details of a relative, friend or neighbour living at a **different address** who we could contact if for any reason we were unable to get hold of you. **Please indicate their relationship to you and write their contact details clearly in the boxes provided.**

Relationship: ☐ **Relative** ☐ **Friend** ☐ **Neighbour** ☐ **Other**

Title: Mr ☐ Mrs ☐ Ms ☐ Miss ☐ Other ☐☐☐☐☐☐☐☐☐☐☐☐☐

First name(s): ☐☐☐☐☐☐☐☐☐☐☐☐☐☐☐☐☐☐☐☐☐☐☐☐☐☐☐

Surname: ☐☐☐☐☐☐☐☐☐☐☐☐☐☐☐☐☐☐☐☐☐☐☐☐☐☐☐

Address: ☐☐☐☐☐☐☐☐☐☐☐☐☐☐☐☐☐☐☐☐☐☐☐☐☐☐☐

☐☐☐☐☐☐☐☐☐☐☐☐☐☐☐☐☐☐☐☐☐☐☐☐☐☐☐

☐☐☐☐☐☐☐☐☐☐☐☐☐☐☐ Postcode: ☐☐☐☐☐☐☐

Telephone number (inc. code): ☐☐☐☐☐☐☐☐☐☐☐☐☐☐☐☐☐☐☐☐☐☐☐☐☐☐☐

9. Continuing in ASCEND

Thank you for completing the questionnaire. If you are happy to continue taking the ASCEND tablets and capsules for the next 5 years, then please SIGN and DATE the form below using blue or black ink, and return it in the FREEPOST envelope provided. Within about 2 weeks of us receiving your questionnaire, if eligible, you will receive a new box of ASCEND medication and will be asked to take one tablet and one capsule daily.

I am happy to take part in ASCEND:

(Please use blue or black ink)

Signature: ________________

& **PRINTED** name: ________________

Today's date: ☐☐ / ☐☐ / 2 0 ☐☐
Day Month Year

Please check that you have answered **every** question, and **signed and dated** the form.
Return the completed questionnaire in the **Freepost** envelope provided **(no stamps needed)** to:-

Freepost RLUJ-TKES-SURB, ASCEND, Richard Doll Building, University of Oxford, Old Road Campus, Headington, Oxford, OX3 7LF

If you have any questions about the study, please contact the coordinating centre in Oxford on **FREEFONE: 0800 585323** (preferably during office hours 9 am - 5 pm, Monday to Friday)

Thank you very much
