# Supplementary material for: Cost-effective recruitment methods for a large randomised trial in people with diabetes: A Study of Cardiovascular Events iN Diabetes (ASCEND)
Source: Trials. 2016 Jun 13;17:286. doi: 10.1186/s13063-016-1354-9 (PMC4907276; doi:10.1186/s13063-016-1354-9)
Supplement: Additional file 6: — ASCEND Study Collaborative Group. (PDF 19 kb) [file 13063_2016_1354_MOESM6_ESM.pdf]

Members of ASCEND Study Collaborative Group

Writing Committee

T Aung, R Haynes, J Barton, J Cox, A Murawska, K Murphy, M Lay, J Armitage, L Bowman

Steering Committee

Chairman: R Collins, *Study coordinator:* J Armitage; *Clinical coordinator:* L Bowman; *Statisticians:* S Parish, R Peto; *Administrative coordinator:* J Barton; *Lay member:* D Simpson; *Other members:* A Adler, T Aung, C Baigent, HJ Bodansky, A Farmer, R Haynes, R McPherson, HAW Neil, P Sleight, P Weissberg.

Data Monitoring Committee

Chair: P Sandercock, *Members:* H Gerstein, R Gray, C Hennekens.

Coordinating Office (Clinical Trial Service Unit, Nuffield Department of Population health, University of Oxford):

Administration and support: J Barton, L Fletcher, K Murphy (coordinators); P Achiri, A Armitage, S Bateman, V Booker, K Brown, F Butcher, E Butler, S Butler, L Cobb, L Cobb, A Collett, P Colmenero, J Crowther, S Fathers, K Frederick, E Goodfellow, E Goodwin, C Hope, A Karnad, V Keyte, M King, S Knight, R Lee, O Machin, N Mohammed, L Pank, A Papadaki, E Pearson-Burton, S Pickworth, A Radley, E Roberts, K Roby, J Sayer, S Smith, S Sutherland, H Thorne, A Timadjer, M Willett, M Wincott, C Wise, J Woods, S Yates.

Clinical support and adjudication: A Alexander, J Armitage, T Aung, S Beebe, L Bowman, L Bromhall, R Bulbulia, F Chen, H Cowan, T Dasgupta, K Gaba, R Haynes, W Herrington, L Hirt, A Isaew, J James, P Judge, A Lawson, D Lewis, R Llewellyn-Bennett, H Lochhead, M Mafham, W Majoni, C Murray, K Naessens, T Porter, K Rahimi, C Reith, E Sammons, B Storey, M Taylor-Clarke, V Toghill, J Tomson, K Walter, E Waters, L Young.

Statistics and computing: P Harding, M Lay, S Parish (coordinators); D Bennett, C Berry, J Booth, Y Bu, G Buck, G Coates, J Cox, M Craig, P Dalton, C Daniels, C Dawe, A Delmestri, A McDougall, Y Mostefai, A Murawska, M Nunn, N Prajapati, W Stevens, S Syed, M Turakani, K Wallendszus, A Young.

CTSU Wolfson Laboratory: S Clark, K Emmens, M Hill, K Kourellias, M Radley, J Wintour (coordinators); M Allworth, L Boggs, T Chavagnon, R Cox, J Cwikowska, T Glass, N Goodwin, A Gordon, J Gordon, C Guest, C Hickman, J Hill, R Hrusecka, N Illingworth, JM Ji, M Lacey, N Luker, K Nafousi, S Norris, N Plunkett, L Sansom, R Shellard, J Taylor, P Taylor, J Wheeler, T Williams, M Yeung.

Collaborators

Addenbrooke's Hospital, Cambridge: A Adler; *Ashford Hospital:* U Meyer-Bothling; *Bolton Diabetes Centre:* J Dean; *Charing Cross Hospital:* J Car, A Dornhorst; *Chesterfield Royal Hospital:* R MacInerney; *City Hospital, Birmingham:* P De; *Colchester General Hospital:* C Bodmer; *NHS Cumbria:* R Wagstaff; *NHS Derby City:* M Browne; *NHS Derbyshire County:* T Humphries; *Dewsbury & District Hospital:* T Kemp; *Frimley Park Hospital, Surrey:* G Menon; *Gloucester Royal Hospital:* T Ulahannan; *Harrogate District Hospital:* P Hammond; *Hemel Hempstead General Hospital:* C Johnston; *Hospital of St Cross, Warwickshire:* JP O'Hare; *Huddersfield Royal Infirmary:* T Burrows, H Griffiths; *Hull Royal Infirmary:* S Atkin, C Walton; *Ipswich Hospital:* P Twomey; *James Cook University Hospital, Middlesbrough:* R Bilous; *Kidderminster Hospital:* P Newrick; *King's Mill Hospital, Nottinghamshire:* I Idris, R Lloyd-Mostyn; *Lavender Hill Group Practice, London:* J Gray; *Leeds General Infirmary:* HJ Bodansky; *Leighton Hospital, Cheshire:* S Mallya; *Lincoln County Hospital:* K Sands; *Macclesfield District General Hospital:* Z Hasan; *Manchester Royal Infirmary:* R Malik; *Newcastle General Hospital:* G Hawthorne, C Jones-Unwin; *Newham University Hospital, London:* S Gelding; *NIHR Clinical Research Network: West Midlands:* M Porcheret; *North Tyneside General Hospital:* S Bennett, N Lewis-Barned; *Peninsula Medical School (Primary Care), Exeter:* P Evans; *Peterborough City Hospital:* S Martin; *Pilgrim Hospital, Lincolnshire:* C Nyman, S Olczak; *Pontefract General Infirmary:* C White; *Poole General Hospital:* A McLeod; *Princess Royal Hospital, Telford:* N Capps; *Queen Alexandra Hospital, Portsmouth:* M Cummings; *Queen's Hospital, Burton-on-Trent:* T Reynolds; *Queen's Medical Centre, Nottingham:* T Gazis; *Rotherham District General Hospital:* R Muthusamy, S Muzulu; *Royal Albert Edward Infirmary Wrightington, Wigan:* S Natha; *Royal Berkshire Hospital:* H Simpson; *Royal Blackburn Hospital:* S Ramtoola; *Royal Bolton Hospital:* A Hutchesson; *Royal Cornwall Hospital:* S Fleming; *Royal Derby Hospital:* GD Tan; *Royal Devon & Exeter Hospital:* K MacLeod (deceased); *Royal Hallamshire Hospital, Sheffield:* C Brand; *Royal Liverpool University Hospital:* D Broadbent, J Vora; *Royal Oldham Hospital:* D Bhatnagar, B Mishra; *Royal United Hospital Bath:* J Reckless; *Sandwell District General Hospital:* E Hughes, D Robertson; *South Tyneside District Hospital:* C Thomas; *St Helier Hospital:* S Hyer, A Rodin; *St James's University Hospital, Leeds:* S Gilbey; *St Mary's Hospital, London Paddington:* D Gable; *St Peter's Hospital, Chertsey:* M Baxter; *Stepping Hill Hospital, Stockport:* P Hale, N Kong, G Burrows; *The Royal London Hospital:* T Chowdhury, D Peterson; *Torbay Hospital:* R Paisey; *University Hospital of Wales:* R McPherson; *Warrington Hospital:* P Chattington; *Watford General Hospital:* M Clements; *Weston General Hospital:* P Singhal; *William Harvey Hospital, Ashford:* A Jafree, C Williams; *Worthing Hospital:* G Caldwell, M Signy; *Wycombe General Hospital:* I Gallen; *York District Hospital:* P Jennings.
